# *QuickStats:* Percentage of Emergency Department Visits for Pain* at Which Opioids^†^ Were Given or Prescribed, by Geographic Region^§^ of the Hospital — United States, 2005–2017

**DOI:** 10.15585/mmwr.mm6902a6

**Published:** 2020-01-17

**Figure:**
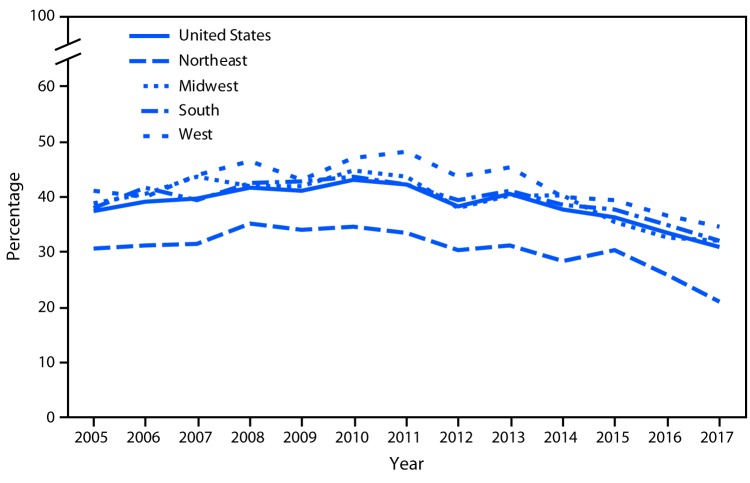
The percentage of ED visits for pain at which an opioid was given or prescribed increased from 37.4% in 2005 to 43.1% in 2010 and then decreased to 30.9% in 2017. A similar pattern was observed in all four regions. Percentages for the Northeast were lower than for the nation as a whole for all years analyzed. In 2017, the percentage was 21.1% in the Northeast, compared with 32.0% in the Midwest, 32.0% in the South, and 34.7% in the West.

**Source:** National Center for Health Statistics. National Hospital Ambulatory Medical Care Survey, 2005–2017. https://www.cdc.gov/nchs/ahcd/ahcd_questionnaires.htm.

